# Interchanges and movements of humpback whales in Japanese waters: Okinawa, Ogasawara, Amami, and Hokkaido, using an automated matching system

**DOI:** 10.1371/journal.pone.0277761

**Published:** 2022-11-17

**Authors:** Nozomi Kobayashi, Satomi Kondo, Koki Tsujii, Katsuki Oki, Masami Hida, Haruna Okabe, Takashi Yoshikawa, Ryuta Ogawa, Chonho Lee, Naoto Higashi, Ryosuke Okamoto, Sachie Ozawa, Senzo Uchida, Yoko Mitani

**Affiliations:** 1 Okinawa Churashima Research Center, Okinawa Churashima Foundation (OCF), Motobu, Okinawa, Japan; 2 Okinawa Churaumi Aquarium, Okinawa Churashima Foundation, Motobu, Okinawa, Japan; 3 Everlasting Nature of Asia (ELNA), Ogasawara Marine Center, Chichi-jima, Ogasawara-mura, Tokyo, Japan; 4 Ogasawara Whale Watching Association, Chichi-jima, Ogasawara-mura, Tokyo, Japan; 5 Amami Whale and Dolphin Association, Naze, Amami, Kagoshima, Japan; 6 Cybermedia Center, Osaka University, Mihogaoka, Ibaraki, Osaka, Japan; 7 Field Science Center for Northern Biosphere, Hokkaido University, Hakodate, Hokkaido, Japan; CMAVE, USDA-ARS, UNITED STATES

## Abstract

Humpback whales in the western North Pacific are considered endangered due to their small population size and lack of information. Although previous studies have reported interchanges between regions within a population, the relationship between the geographic regions of a population in Japan is poorly understood. Using 3,532 fluke photo IDs of unique individuals obtained from four areas in Japan: Hokkaido, six IDs (2009–2019); Ogasawara, 1,477 IDs, from two organizations (1) Everlasting nature of Asia (1987–2020) and (2) Ogasawara Whale Watching Association, (1990–2020); Amami, 373 IDs (1992–1994, 2005–2016); Okinawa, 1,676 IDs (1990–2018), interchanges were analyzed. The ID matchings were conducted using an automated system with an 80.9% matching accuracy. Interchange and within-region return indices were also calculated. As a result, number of matches and interchange indices follow locations, Hokkaido-Okinawa (3, 0.31), Amami-Ogasawara (36, 0.06), Amami-Okinawa (222, 0.37), and Okinawa-Ogasawara (225, 0.08), respectively. Interchange indices among Japanese areas were much higher than the indices between Ogasawara/Okinawa and Hawaii (0.01) and Mexico (0.00) reported in previous studies, indicating that the Japanese regions are utilized by the same population. At the same time, the frequency of interchanges among the three breeding areas vary, and the high within-region return indices in respective breeding areas suggest the site fidelity of the whales in each area at some level. These results indicate the existence of several groups within the population which are possibly be divided into at least two groups based on geographical features: one tend to utilize Ogasawara and the Mariana Archipelago; the other utilize Amami, Okinawa, and the Philippines, migrating along the Ryukyu and Philippine Trench. The matching results also suggest that Hokkaido is possibly be utilized as a corridor between northern feeding areas and southern breeding areas at least by individuals migrating to Okinawa area.

## Introduction

Humpback whales (*Megaptera novaeangliae*) are widely distributed in all major oceans and migrate seasonally between high-latitude feeding areas and low-latitude breeding areas [1, 2]. In the North Pacific, feeding aggregations of humpback whales occur in waters from California to Russia during summer [3, 4]. In winter, they migrate to breeding areas in the coastal waters of Mexico and the Revillagigedo Archipelago [5], the Hawaiian Islands [6], Japan [7–9], the Philippines [10], and the Mariana Archipelago [11].

Humpback whales were listed as an endangered species globally under the US Endangered Species Act in 1970 because of the severe population decline caused by commercial whaling worldwide including the areas around Japan [12] until 1966, when the International Whaling Commission prohibited the commercial hunting of humpback whales. It has been suggested that the humpback whale population worldwide has subsequently begun to increase [13]. In 2016, the US National Marine Fisheries Service reassessed humpback whales and reclassified the population into 14 distinct population segments (DPS) globally, some of which were delisted where adequate population recovery had been confirmed [14]. However, in the North Pacific, the western North Pacific, and Central America subpopulations are still listed as endangered because of the lack of information and insufficient population recovery. Meanwhile, the subpopulation in Mexico is considered ‘threatened’ and the Hawaiian subpopulation does not require conservation [14, 15]. From 2004–2006, an international collaborative study on humpback whales throughout the North Pacific, called ‘Structure of Populations, Levels of Abundance and Status of Humpbacks’ (SPLASH) was conducted. The study suggests that the population structure of the North Pacific humpback whales is complex [3]. In addition, there are many questions left regarding certain relationships, especially among the sighting areas in the western North Pacific, since there was a limited amount of data obtained in the region at that time. This is one of the main reasons why the International Union for Conservation of Nature (IUCN) is concerned about the western North Pacific population [15, 16], and the whales are classified as endangered in the US [14].

In the western North Pacific, waters around Ogasawara, Okinawa, Amami-Oshima (Amami) in Japan, the Philippines, and Mariana Archipelago are known as breeding areas of humpback whales [8–11, 17–19]. Previous studies have demonstrated that the breeding areas in the western North Pacific are likely to share the same population because the same individuals of humpback whale are observed among the different regions, such as between Ogasawara and Okinawa [20], the Philippines and both Ogasawara [21] and Okinawa [10, 22], as well as the Mariana Archipelago and the other regions [11]. There was a significant difference in mitochondrial (mt-)DNA haplotype frequencies between Ogasawara and both Okinawa and the Philippines [16], as well as between the Mariana Archipelago and both Okinawa and the Philippines, while no significant differentiation was found between Mariana Archipelago and Ogasawara frequencies [11]. Based on these results, the authors postulated the existence of several subpopulations in the western North Pacific. Acebes et al. [10] also noted that the breeding areas in the western North Pacific were likely to share the same population and that whales also exhibited some degree of fidelity to their respective breeding areas. In addition, possibly related to the increasing trend of the entire North Pacific population [13], humpback whale sightings in several new sighting areas in Japan have also been reported in recent years, such as in Mikura Island [23], Hachijo Island [24], and Yakushima Island [25], which are located in the potential migratory route between the known feeding and breeding areas in the western North Pacific.

In Japan, sighting surveys on humpback whales based on photo identification have been regularly conducted since the 1980s in Ogasawara [7], and the early 1990s in Amami, and Okinawa [17]. The location and water temperature of the known winter breeding areas worldwide are reported in warm waters with sea-surface temperatures ranging from 21.1–28.38°C (average 24.68°C), irrespective of latitude [26]. The sea-surface temperatures in Ogasawara, Amami, and Okinawa were well within the reported range. In addition, in these areas, a number of mother and calf pairs as well as mature individuals are repeatedly observed every year, not only passing through these areas as corridors, but also staying and utilizing these areas for more than a few days during the breeding season. Competitive groups of more than three whales (which are considered as groups with multiple males competing over females for mating chances [27, 28]), have also been observed in these areas. Furthermore, the majority of whales in these areas are distributed primarily in water depths of < 200 m [9, 17, 20], which is characteristic of the humpback whale breeding habitat [11, 29]. On behalf of all these considerations, these three areas are considered to be breeding areas for humpback whales.

Currently, sighting records of humpback whales have also been obtained from the southeastern offshore waters of Hokkaido. The same individual had been observed both in Hokkaido and Okinawa [30] suggesting that the Hokkaido area is a migratory corridor of humpback whales between northern feeding areas and southern breeding areas and/or are potential feeding areas for the species. There have been no studies to date conducted on the interchanges of humpback whales among all of these regions in Japan. The large amount of data obtained in each region in the past decade has made it difficult to conduct the interchange analysis using photo identification in all regions at once. However, it is crucial to understand the relationship between these sighting regions in Japanese areas to understand the clear population structure in the western North Pacific, which is vague at present.

In this study, the interchanges and movements between four humpback whale sighting regions in Japan, Hokkaido, Ogasawara, Amami, and Okinawa were analyzed for the first time with the results of an automated fluke matching system using the most recent fluke ID catalogs in each region. The timing and movement patterns of humpback whales within and across seasons were also investigated. This study aims to clarify the relationship between these regions to obtain information that is useful for the conservation and management plans of humpback whales in the western North Pacific population.

## Material and methods

### Four sighting regions in Japan

**Fig 1:**
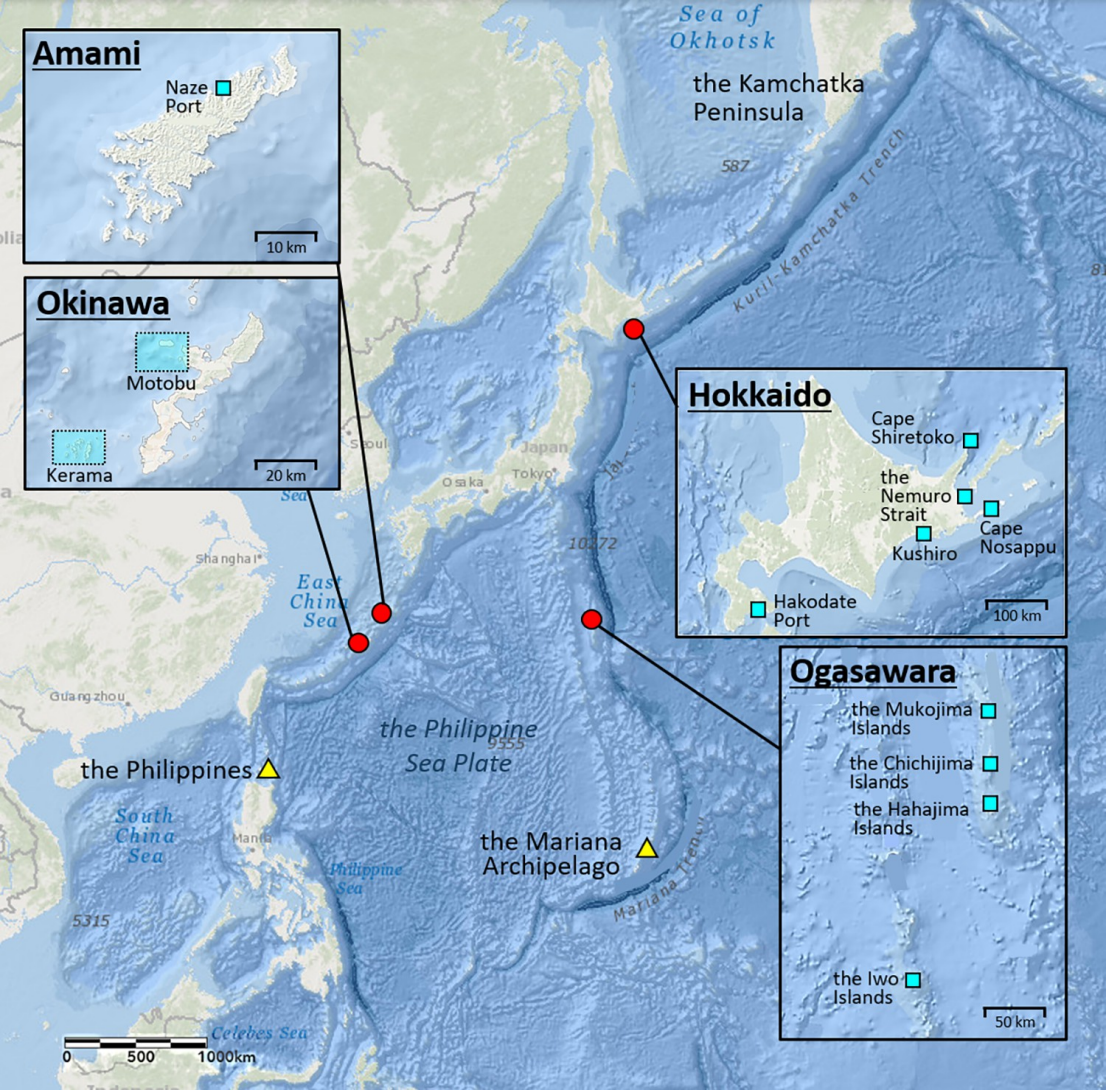
Locations of humpback whale survey areas in the western North Pacific. The blue squares in the enlarged maps represent the survey areas and/or departure ports of the survey vessels in each area in Japanese waters: Hokkaido, Ogasawara, Amami and Okinawa. The map image is published from PLOS ONE under a CC BY license, with permission from Environmental Systems Research Institute, Inc. (Esri), original copyright 2022, also the content is the intellectual property of Esri and is used herein with permission. Copyright © 2022 Esri and its licensors. All rights reserved.

The data used for this study were obtained from five independent research organizations in four different regions in Japan: Hokkaido, Ogasawara, Amami, and Okinawa (Fig 1). Three of these regions, Ogasawara, Amami, and Okinawa are known as breeding areas for humpback whales, and whales have been observed annually during the breeding season, mainly from December to May [7, 9, and 17].

Humpback whales have also been observed off the southeastern coast of the Hokkaido area in October [30]. The waters off Hokkaido are considered to be either a migration route from the northern feeding areas to the southern Asian breeding areas or a possible feeding area for humpback whales in the western North Pacific. The survey area, period, and data collection methodologies for the four areas in this study are described in detail below.

### Survey areas in each region

In Hokkaido, sighting survey vessel departed from Hakodate (41° 46′ 53′′ N, 140° 42′ 18′′ E) and traveled along the Pacific coast of eastern Hokkaido, including Cape Nosappu (43° 23′ 07′′ N, 145° 49′ 01′′ E) to the Nemuro Strait, and Cape Shiretoko (44° 20′ 43′′ N, 145° 19′ 47′′ E) to the Sea of Okhotsk (Fig 1). In Ogasawara, surveys were conducted by two independent research organizations: the Ogasawara Marine Center, Everlasting Nature of Asia (ELNA), and Ogasawara Whale Watching Association (OWA). Surveys by ELNA and OWA were conducted in four areas: around the Chichijima Islands (27° 05′ 50′′ N, 142° 12′ 02′′ E), the Hahajima Islands (26° 38′ 11′′ N, 142° 09′ 30′ E), the Mukojima Islands (27° 40′ 43′′ N, 142° 08′ 09′′ E), and the Iwo Islands (24° 48′ 11′′ N, 141° 18′ 04′′ E) (Fig 1), when surveys around the waters of the Iwo Islands were conducted only from 1993–1998 by ELNA. In Amami, experienced researchers boarded the whale-watching vessels of the members of the Amami Whale and Dolphin Association and conducted surveys. The vessels mainly departed from Naze port and surveys were conducted all around Amami-Oshima Island. Okinawa surveys were conducted off the coast of the Motobu Peninsula and around Ie Island (study area indicated as Motobu, 26° 33′ 12′′ N, 127° 34′ 22′′ E–26° 48′ 37′′ N, 127° 56′ 11′′ E), and the Kerama Islands (study area indicated as Kerama, 26° 03′ 38′′ N, 127° 06′ 25′′ E–26° 24′ 14′′ N, 127° 31′ 10′′ E) (Fig 1).

### Data used in the study

In this study, total of 3,532 fluke photo IDs of unique individuals obtained in four regions were used for the analysis (Table 1). Surveys in each region were conducted by survey vessels on weather permitting days (Beaufort scale < 5 and visibility > 1 km) by at least two observers. When whales were sighted, the sighting location (latitude and longitude), time, group size, and group composition were recorded, and when possible, photographs of the ventral side of the tail flukes were collected. Photographs were taken using single-lens reflex cameras (e.g., Canon EOS 80D; Canon Inc., Ohta-ku, Tokyo) with fixed zoom lens (e.g., Canon EF-S18-200 mm; Canon Inc., Ohta-ku, Tokyo, Japan), and the locations were recorded using global positioning system (GPS) receivers (e.g., GARMIN Geko 201; GARMIN International, Inc., Olathe, KS, USA). In addition, some of the fluke photographs taken between November and May from 1990 to 2020 in Ogasawara, between January and May from 1992 to 2016 in Amami, and between January and April from 1990 to 2018 in Okinawa were provided by citizen scientists. The presence of singers in the groups was checked using hand-held hydrophones (e.g., AQH-020; Aquafeeler IV; AquaSound Inc., Chuo-ku, Koube, Japan). The sex of individual whales was determined by their social roles based on the definitions described by Darling et al. [31] and Glockner [32] as follows:

Male: A single individuals escorting mothers and calves or individuals that were confirmed to be singing (singer) during the surveys [31, 32].

Female: Individuals closely associated calves during the surveys [32].

**Table 1:** Study years, periods, vessels, and number of individuals (IDs) obtained from each of the sighting region.

Region	Years	Periods	Survey	No. of individuals(IDs)	Research organization
			Vessels		
Hokkaido	2009–2019	Sep-Oct	T/S Ushio-maru of Hokkaido University (286 gross tonnage)	6	Field Science Center for Northern Biosphere, Hokkaido University
Ogasawara	1987–2014, 2017–2020	Dec-May	6-7m long	1246*	Ogasawara Marine Center, Everlasting Nature of Asia (ELNA)
			research vessels		
Ogasawara	1990–2020	Nov-May	5.71m long	385*	Ogasawara Whale Watching Association (OWA)
			research vessel		
Amami	1992–1994, 2005–2016	Jan-Apr	local whale-watching	373	Amami Whale and Dolphin Association
			vessels		
Okinawa	1990–2018	Jan-Apr	3.2–4.9 ton	1676	Okinawa Churashima Research Center, Okinawa Churashima Foundation (OCF)
			research vessels		
Total				3532	

* Combined number of IDs for Ogasawara were 1477 from ELNA and OWA without the duplication of same individuals in the catalogs

### Data analysis

#### Photo-identification matching

Humpback whales can be identified individually from photographs of the black and white color patterns and the shape of the edges on the ventral surface of the flukes [33]. Photographs of the ventral side of flukes of humpback whales (IDs) collected in each area were compiled into a catalog for each organization following an established method and process [3]. For Ogasawara, two organizations conducted humpback whale surveys in the same study area. Therefore, two different ID catalogs exist for the region, one for ELNA and the other for OWA. These two different catalogs were compared with each other using the established method and process by experienced researchers [3] and combined as one catalog for the Ogasawara area by integrating the duplication of the same whales in both catalogs.

Comparisons of the catalogs between four different areas were conducted to analyze the interchange between the four areas using an automated fluke matching system developed by Osaka University, Diagence corp. (Tokyo, Japan), Keio University, and OCF with a combination of rough detection of deep learning and precise decisions of image processing [34]. The best quality ID photo for each unique whale in each ID catalog was put into the matching system. Then, the IDs from each area were compared with each other using all possible regression methods to find the same whales in different areas. The system provides a list with 20 unique IDs that were detected as possible matches for each input ID. Then, the experienced researchers in each organization confirmed if there were IDs for the same whales within the top 20 listed as unique IDs in the list. The matching accuracy for the system was 80.9% when the analog matchings by experienced researchers was considered to be 100% accuracy. When matches of the same individuals were found between two different regions, they were defined as an interchange between these waters, and the results were used for further analysis. The sighting histories (date, location, and group composition) of the individual in each area were compared to analyze whether the individual moved between the regions within a year or across years.

### Interchange and within-region return indices

To analyze and compare the frequency of the movements within and among the regions, the interchange index between different regions and within-region return index in each region was calculated by following the methodologies of Calambokidis et al. [6], Garrigue et al. [35], Urban et al. [36], and Acebes et al. [10]. Interchange indices were calculated using all the best photo IDs obtained through the whole survey years in each region. Within-region return indices in this study were calculated for the common three latest years (i.e., 2014, 2015, and 2016) for the four regions in the respective catalogs. The interchange index was calculated as follows:

$$Interchange\;Index=M_{1,\;2}/(N_{1}*N_{2})\;*\;1000$$ (1)

where

N_1_ = number of whales identified in region 1 (e.g., Okinawa),

N_2_ = number of whales identified in region 2 (e.g., Ogasawara),

M_1_, _2_ = number of whes re-sighted in both regions.

The within-region return index was calculated as:

Within‐regionreturnindex=Mi,j/(Ai*Bj)*1000 (2)

where

Ai = the number of whales identified in all the years before the study period (e.g., 1990–2013) in a target region (e.g., Okinawa),

Bj = the number of whales identified during the study period (e.g., 2014) in a target region (e.g., Okinawa),

Mi, j = the number of whales marked in any previous year, and re-sighted in the study year (e.g., 2014) in a region (e.g., Okinawa).

Both interchange and within-region return indices were calculated to be zero when there was no re-sighting of whales [35]. A high interchange index indicates a high probability that whales move actively between the areas and a high within-region return index indicates a high probability that the same individual would be re-sighted in the same area (i.e., the result of a small population being present, or high connectivity) [10]. Conversely, a low interchange index indicates that whales do not migrate between areas often and a low within-region return index indicates that either the population in an area is large or the whales actively move between the areas [6]. The interchange indices between the Mariana Archipelago and Okinawa, Ogasawara, and the Philippines were also calculated using the data presented by Hill et al. [11] using the same formulas (Eq 1 and Eq 2) to analyze the relationships between the breeding areas in the wider areas of the western North Pacific.

### Within-season movements

The date of sightings were reviewed for the individuals that were identified in two different regions in the same season to analyze the movement between regions in a year. The duration of migration between areas by the same whales was estimated from the first and last days when whales were observed in each area. The sex of the matched individuals between the two different areas was also analyzed to study the trend of movement in the respective sexes.

### Statistical analysis

To analyze the interchange indices along with the distances between the sighting areas, the indices were standardized based on the following equation (Eq 3). The correlation coefficient between the distances and standardized indices was then calculated to analyze the correlation between the distance among the areas and the indices. All statistical analyses were performed using Microsoft Excel 2019 (Microsoft Corp., Redmond, WA, USA).

Standardizeddifference=(X‐m)/σ (3)

where

X = interchange indices,

m = average

σ = standard deviation

## Rults

### Matching results of photo identification between the regions

Overall, 486 matches were confirmed among the four regions: three matches (i.e., three of unknown sex) between Hokkaido and Okinawa; 36 matches (i.e., 12 males, one female, and 23 of unknown sex) between Ogasawara and Amami; 225 matches (i.e., 88 males, 23 females, and 114 of unknown sex) between Ogasawara and Okinawa; 222 matches (i.e., 45 males, 29 females, and 148 of unknown sex) between Amami and Okinawa (Table 2). No matches were found between Hokkaido and Ogasawara or Amami in this study.

**Table 2:** Interchange indices, proportions (%) and the actual number of matched individuals in two different regions.

Region (A)	Region (B)	Interchange index	No. of individuals resighted in both regions (Sex of the matched individuals)*	% of No. of individuals in region (A) which resighted in both areas	% of No. of individuals in region (B) which resighted in both areas	Survey years in region (A)	Data source
Hokkaido	Ogasawara	**0**	0	0	0	2009–2019	This publication
	Amami	**0**	0	0	0		
	Okinawa	**0.31**	3 (M:0, F:0, U:3)	50.00	0.17		
Ogasawara	Amami	**0.06**	36 (M:12, F:1, U:23)	2.43	9.65	1987–2020	
	Okinawa	**0.08**	225 (M:88, F:23, U:114)	15.23	13.42		
Amami	Okinawa	**0.37**	222 (M:45, F:29, U:148)	59.51	13.81	1992–1994, 2005–2016	
Okinawa	-	**-**	-	-	-	1990–2018	
Total			486				
Mariana Archipelago	Ogasawara	0.10	7 (M:2, F:3, U:2)	17.07	0.35	2015–2018	Hill et al. 2020 [11] Interchange indexes and percentages were calculated from the numbers in Hill et al. 2020 [11]
	Okinawa	0.06	4 (M:3, F:1, U:0)	9.75	0.24		
	the Philippines	0.10	1 (M:1, F:0, U:0)	2.43	0.42		
the Philippines	Ogasawara	0.27	86 (M:40, F:14, U:32)	36.80	6.20	1999–2016	Acebes et al. 2021 [10]
	Okinawa	0.30	100 (M:41, F:26, U:33)	43.48	6.92		
Hawaii	Ogasawara	0.01	4	0.34	1.55	1991–1993	Calambokidis et al. 2001 [6], Darling and Cerchio. 1993 [37], Salden et al. 1999 [38]
	Okinawa	0	0	0	0		
Mexico	Ogasawara	0	0	0	0		
	Okinawa	0	0	0	0		

* M = male, F = female, U = Unknown

The match rate among the four regions was highest for Amami with Okinawa (59.51%), followed by Hokkaido with Okinawa (50.00%). The lowest rate was confirmed for Hokkaido and both Ogasawara and Amami with zero matches, followed by Okinawa with Hokkaido (0.17%), and Ogasawara with Amami (2.43%) (Table 2).

**Fig 2:**
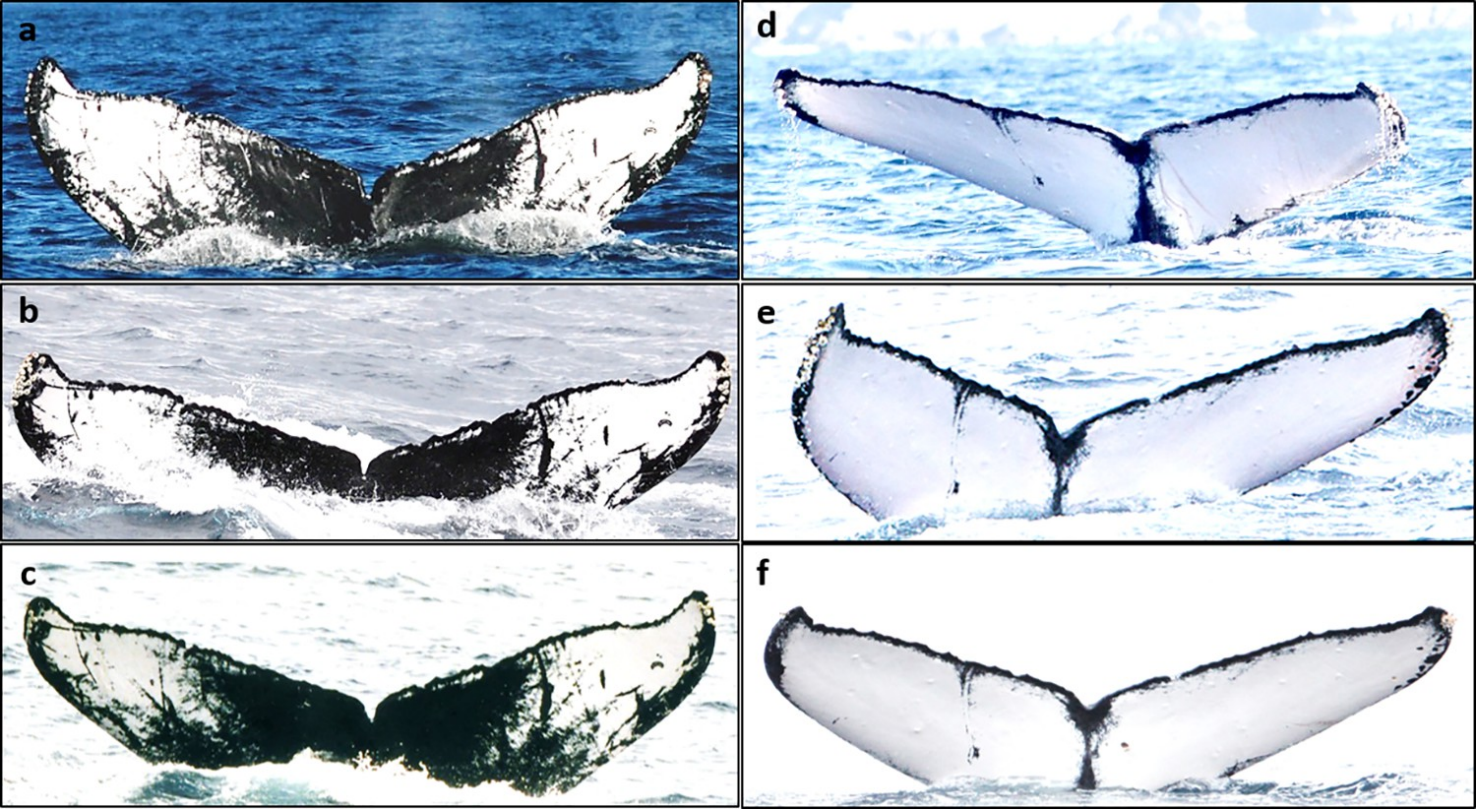
Examples of matched individuals among the three breeding areas in Japan. (a) Individual ‘OG-MN14/O-54’ observed in Ogasawara. (b) The same individual, ‘A-157,’ observed in Amami, and (c) ‘R-7,’ also the same individual, observed in Okinawa. (d) Individual ‘O-5’ observed in Ogasawara. © Same individual, ‘A-171,’ observed in Amami, and (f) ‘R-467’ also the same individual observed in Okinawa. The fluke images are published from PLOS ONE under a CC BY license, with permissions from Ogasawara Whale Watching Association for image (a), Everlasting nature of Asia for image (a), and (d), Amami Whale and Dolphin Association for images (b), and ©, and Okinawa Churashima Research Center for images (c), and (f), all original copyright 2022.

There were total of 20 individuals (11 males, two females, and seven of unknown sex) which observed in all of the three sighting areas, Ogasawara, Amami, and Okinawa, in different years (Table 3, Fig 2 and S1 Table). Of these, some individuals, such as “OG-MN14/O-54; A-157; R-7” and “OG-MN5/O-45; A-287; R-262” were more likely to migrate to either Ogasawara or Okinawa (Table 3 and S1 Table). In contrast, some of them, such as “O-5; A-171; R-467” and “OG-MN62/O-331; A-3; R-114” migrated almost same number of times to both Ogasawara and Okinawa. Overall, fewer whales were seen in Amami and also in other locations across years (Table 3 and S1 Table). Meanwhile, the sex of individuals found in more than two different areas within or among the years was more likely to be male than female (Table 2). It should also be noted, however, that approximately half of the individuals in each area were of unknown sex.

**Table 3:** Number of sightings of humpback whales which were observed in three different sighting areas (Ogasawara, Amami, and Okinawa) in the different years.

#	Whale ID			Number of sightings among different years				Sex*
	Ogasawara	Amami	Okinawa	Ogasawara	Amami	Okinawa	Total	
1	O-5	A-171	R-467	7	1	9	**17**	M
2	OG-MN14/O-54	A-157	R-7	10	1	5	**16**	M
3	OG-MN5/O-45	A-287	R-262	2	1	10	**13**	U
4	O-1171	A-155	R-261	1	1	8	**10**	M
5	OG-MN507/O-1284	A-284	R-213	2	1	7	**10**	M
6	O-904	A-201	R-1004	4	1	5	**10**	M
7	OG-MN62/O-331	A-3	R-114	4	2	4	**10**	M
8	OG-MN216/O-1071	A-218	R-1236	6	1	2	**9**	M
9	O-1200	A-209	R-1166	1	2	5	**8**	U
10	OG-MN416/O-1507	A-221	R-1280	3	1	4	**8**	M
11	OG-MN498	A-54	R-440	1	1	5	**7**	M
12	O-530	A-132	R-1405	5	1	1	**7**	M
13	O-1523	A-60	R-107	1	1	4	**6**	M
14	OG-MN223	A-315	R-993	1	1	4	**6**	U
15	O-1408	A-213	R-1198	2	1	3	**6**	F
16	O-1309	A-341	R-1568	2	1	2	**5**	U
17	O-1547	A-115	R-1316	1	1	2	**4**	F
18	O-620	A-237	R-1480	2	1	1	**4**	U
19	OG-MN431	A-225	R-1324	1	1	1	**3**	U
20	O-502	A-371	R-1722	1	1	1	**3**	U

* M = male, F = female, U = unknown

### Interchange and within-region return indices

Interchange indices were highest between Amami and Okinawa (0.37), followed by Hokkaido and Okinawa (0.31). In contrast, the indices between Ogasawara and the other regions in Japan were relatively low, with all the values being lower than 0.10 (Table 2). The interchange indices between Hokkaido and both Ogasawara and Amami were zero, since there were no matches confirmed between the regions (Table 2).

**Fig 3:**
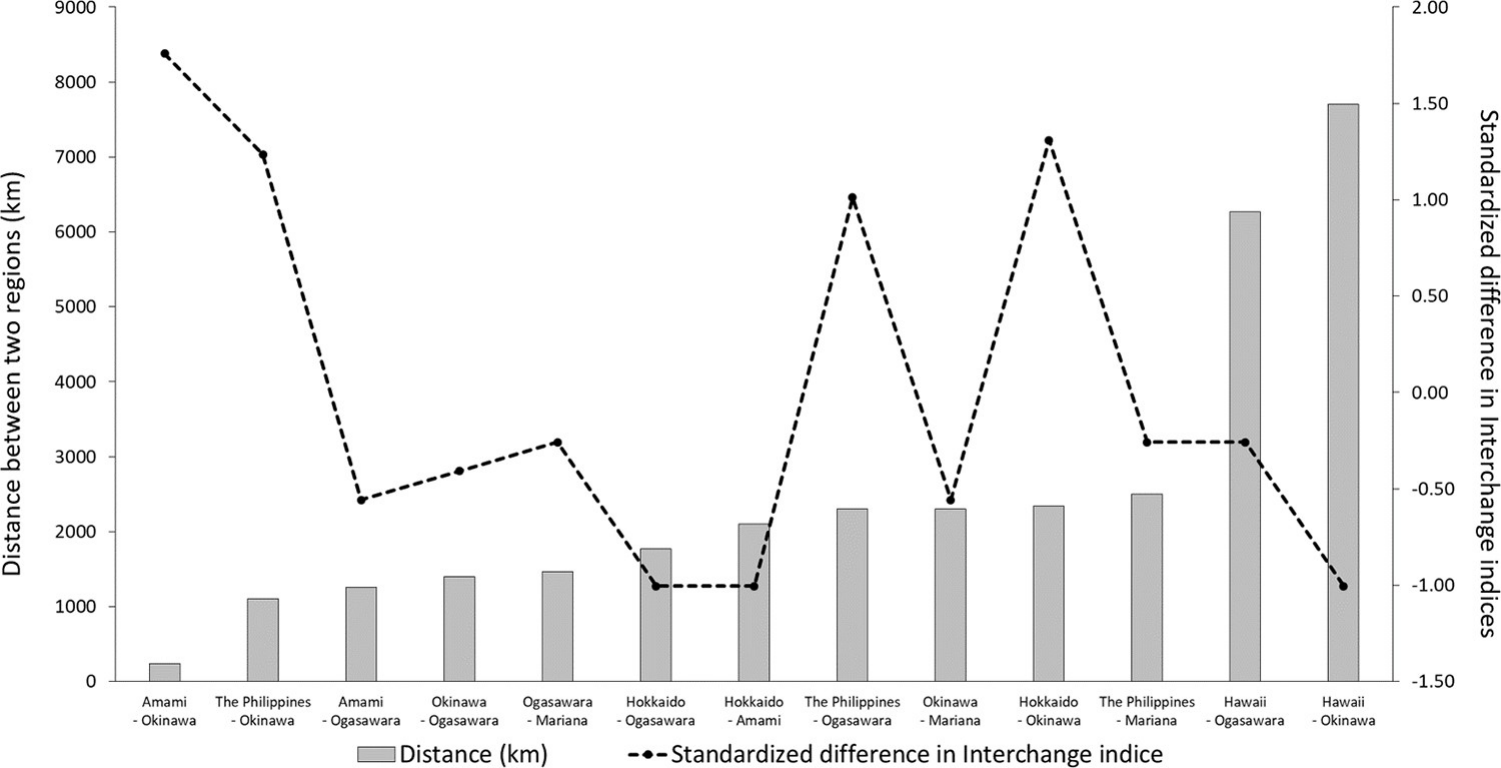
Distance (km) and standardized difference in interchange indices between sighting areas of humpback whales in the western North Pacific. The bar chart and left axis indicate the distance between two sighting areas, when the line chart and right axis indicate the standardized difference in interchange indices between two sighing areas.

Interchange indices between the Mariana Archipelago and both Ogasawara and Okinawa were calculated from the information reported by Hill et al. [11]. The indices between the Mariana Archipelago and Ogasawara (0.10) were slightly higher than those for Okinawa (0.06) (Table 2). The correlation between the standardized differences in interchange indices and the distances between sighting areas in the western North Pacific, including the values shown in previous studies, was calculated to be -0.33, indicating almost no correlation between the distance and the interchange indices among the sighting areas (Fig 3 and S2 Table).

Within-region return indices were calculated for the same last three years in the four regions. The average values for the within-region return indices for multiple years for Hokkaido, Ogasawara, Amami, and Okinawa were 0.00, 0.40, 0.88, and 0.56, respectively (Table 4). Within-region return indices for three breeding areas, Ogasawara, Amami, and Okinawa were relatively higher than the interchange indices between other regions.

**Table 4:** Within-region return indices and the survey years for each of the sighting regions.

Region	Within-region return index	Survey years
Hokkaido	0	2009–2014
	0	2009–2015
	0	2009–2016
Average	**0**	
Ogasawara	0.47	1991–2014
	0.32	1991–2015
	0.41	1991–2016
Average	**0.40**	
Amami	0.94	1992–2014
	0.91	1992–2015
	0.8	1992–2016
Average	**0.88**	
Okinawa	0.58	1991–2014
	0.58	1991–2015
	0.53	1991–2016
Average	**0.56**	

### Within season movements between the regions

A total of 114 cases with 100 unique individuals (i.e., 35 males, 16 females, and 49 of unknown sex) were observed in two different regions within the same season among Ogasawara, Amami and Okinawa (Table 5).

**Table 5:** Within-season movements of whales between Ogasawara, Amami and Okinawa.

Regions (A)-(B)	No. of cases (No. of individuals)	Sex* of unique individuals	Direction and frequencies of movements (No. of individuals), [No. of individuals in each sex]					No. of days between observations		
			(A)→(B)	(B)→(A)	(A)→(B)→(A)	(A)→(B)→(A)→(B)	Unknown	Min.	Max.	Ave.
Ogasawara-Amami	1 (1)	M: 0, F: 1, U: 0	1 (1) [M:0, F: 1, U: 0]	0 [M:0, F: 0, U: 0]	0	0	0	16	16	-
Ogasawara-Okinawa	13 (11)	M: 6, F: 3, U: 2	2 (2) [M:1, F: 1, U: 0]	11(9) [M:5, F: 2, U: 2]	0	0	0	21	100	42.3
Amami-Okinawa	100 (88)	M: 29, F: 12, U: 47	43 (41) [M:16, F: 4, U: 21]	48 (46) [M:11, F: 11, U: 24]	7 (7) [M: 1, F: 1, U: 5]	1 (1) [M: 1, F: 0, U:0]	1(1) [M: 0, F: 0, U: 1]	4	69	16.9
**Total**	114 (100)	M: 35, F: 16, U: 49								

* M = male, F = female, U = unknown

Only one individual (a female) was observed at both Ogasawara and Amami within the same season (Tables 5 and 6). Thirteen cases with 11 unique individuals (i.e., six males, three females, and two of unknown sex) were observed at both Ogasawara and Okinawa in the same season (Tables 5 and 6). Meanwhile, 100 cases with 88 unique individuals (29 males, 12 females, and 47 of unknown sex) were observed both in Amami and Okinawa in the same year (Table 5). Multiple within-season movements between several areas by the same individuals were only documented between Amami and Okinawa in this study for eight cases with eight unique individuals (i.e., two males, one female, and five of unknown sex; Tables 5 and 7). Overall, within the 100 matched individuals that were observed in two different regions among three of the areas within the same season, males were observed more than twice than the females, except for the case between Ogasawara and Amami where only one female was observed in both areas in the same season (Table 5).

**Table 6:** Details of whales observed between Ogasawara, Amami, and Okinawa within the same season. The lines in gray are the westward movements from Ogasawara to Amami/Okinawa, while others are eastward movements from Amami/Okinawa to Ogasawara.

Whale ID			Sex*^1^	Directionality*^2^	Year	Date of observations*^3^		No. of days between the observations
Ogasawara	Amami	Okinawa				First area	Second area	
O-320	-	R-12	U	OK→OG	1992	Mar. 11	Apr. 1	21
OG-MN357/O-465	-	R-31	F	OK→OG	1993	Mar. 21	Apr. 12	22
O-499	-	R-2	M	OK→OG	1993	Feb. 14	Mar. 21	35
	-			OK→OG	1994	Mar. 16	Apr. 11	26
	-			OK→OG	2004	Feb. 19	Apr. 6	47
O-59	-	R-105	M	OK→OG	1994	Feb. 23	Apr. 4	40
O-814	-	R-99	F	OK→OG	1997	Mar. 18	Apr. 15	28
OG-MN7/O-11	-	R-237	M	OK→OG	1998	Feb. 27	Apr. 6	38
OG-MN133/O-1174	-	R-303	M	OK→OG	2002	Feb. 14	Mar. 21	35
O-570	-	R-770	M	OK→OG	2008	Feb. 4	Apr. 28	84
OG-MN291	-	R-1565	U	OK→OG	2015	Jan. 24	May. 4	100
O-551	-	R-37	F	OG→OK	1994	Jan. 8	Feb. 17	40
O-464	-	R-136	M	OG→OK	1996	Jan. 22	Feb. 25	34
OG-MN227/O-1531	A-12	-	F	OG→A	2008	Apr. 4	Apr. 20	16

*1 M = male, F = female, U = Unknown, *2 OG = Ogasawara, A = Amami, OK = Okinawa, *3 Dates of the last and first observations in the first and second area, respectively.

**Table 7:** Details of movement of whales between Amami and Okinawa observed for multiple times within the same season. Numbers in parenthesis are the number of days between the observations within the same area (Okinawa).

Whale ID		Sex*^1^	Directionality*^2^	Year	Location*^2^	Date	No. of days between the observations
Amami	Okinawa						
A-98	R-411	M	A→OK→A	2014	A	Feb. 17	-
					OK	Feb. 24	7
					OK	Mar. 12	(16)
					OK	Mar. 26	(14)
					A	Apr. 3	8
A-5	R-146	F	A→OK→A	2015	A	Jan. 25	-
					OK	Jan. 29	4
					OK	Feb. 1	(3)
					A	Feb. 24	23
A-15	R-198	M	A→OK→A→OK	2015	A	Feb. 7	-
					OK	Feb. 20	13
					OK	Feb. 24	(4)
					A	Mar. 5	9
					OK	Apr. 5	31
A-209	R-1166	U	A→OK→A	2015	A	Feb. 24	-
					OK	Feb. 28	4
					A	Mar. 23	23
A-224	R-1313	U	A→OK→A	2015	A	Feb. 25	-
					OK	Mar. 13	16
					OK	Mar. 14	(1)
					A	Mar. 23	9
A-242	R-1567	U	A→OK→A	2015	A	Feb. 2	-
					OK	Feb. 13	11
					A	Mar. 21	36
A-264	R-1610	U	A→OK→A	2015	A	Feb. 12	-
					OK	Mar. 8	24
					A	Mar. 31	23
A-310	R-921	U	A→OK→A	2016	A	Jan.31	-
					OK	Feb. 22	22
					A	Mar. 16	23

*1 M = male, F = female, U = unknown, *2 A = Amami, OK = Okinawa

Among the whales that moved between different regions within the same season, the duration of travel between the regions varied. Within-season movement between Ogasawara and Amami was confirmed in one case, and the duration of the whale traveling between the areas was 16 d (Tables 5 and 6). In the case of Ogasawara and Okinawa, the duration of travel between the two regions ranged between 21 and 100 d, with an average of 42.3 d (Tables 5 and 6), while it ranged between four and 69 d, with an average of 16.9 d between Amami and Okinawa (Table 5).

**Fig 4:**
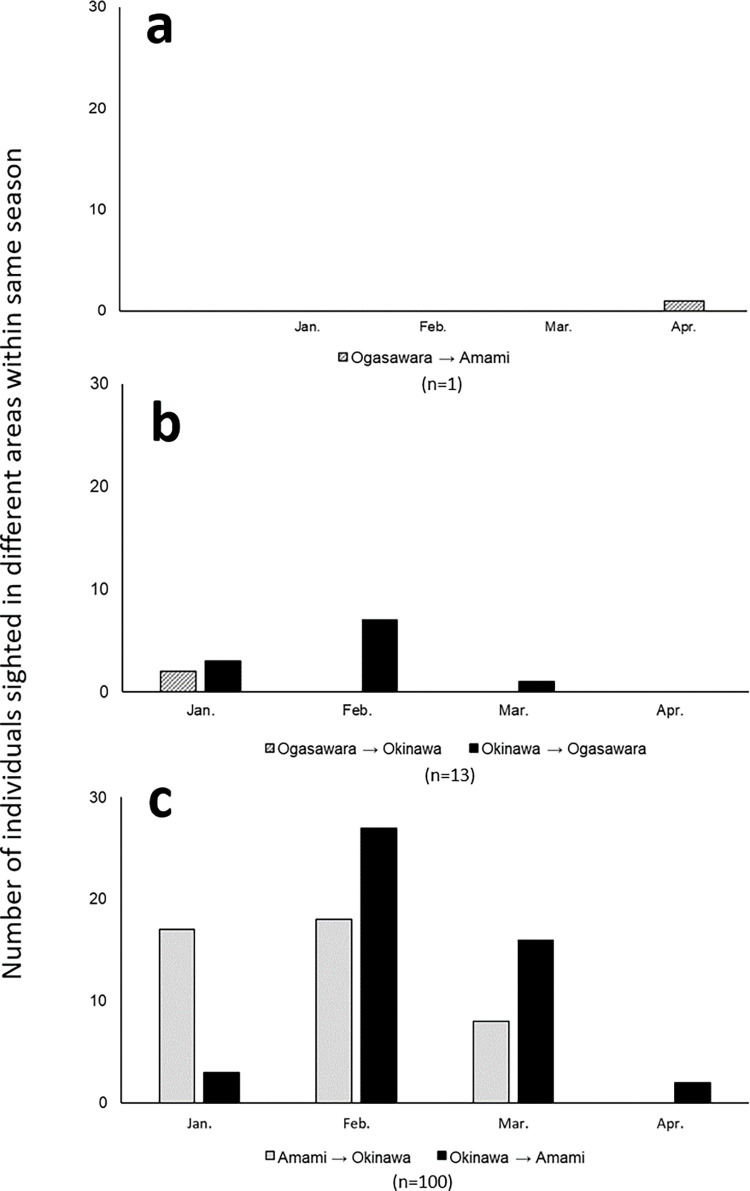
Directions of within-season movements between Ogasawara, Amami, and Okinawa. Each bar represents the number of individuals observed in the first region. These individuals are then observed in the other region later on in the season.

As for the direction and timing of the migration, in the case of Ogasawara and Amami, a whale first seen in Ogasawara in the beginning of April, moved and was observed in Amami in late April, traveling from east to west in the late breeding season (Table 6, Fig 4A and S3 Table).

Between Ogasawara and Okinawa, there were two cases with two individuals observed earlier in Ogasawara in January and then observed later in Okinawa in February, traveling from east to west, from the beginning to the middle of the breeding season (Tables 5 and 6, Fig 4B and S3 Table). In contrast, there were 11 cases with nine individuals observed earlier in Okinawa in January and February, and then observed in the latter months in Ogasawara, traveling from west to east (Tables 5 and 6, Fig 4B and S3 Table). Among them, an individual, ‘O-499/R-2,’ was observed moving between the areas three times in different years, where all the cases were confirmed as eastward movements from Okinawa to Ogasawara. In the case between Amami and Okinawa, southward movement was confirmed in 43 cases, with 41 individuals moving from Amami to Okinawa. Cases were confirmed most often in February and January in Amami, in the early and middle stages of the breeding season (Table 5 and 7, Fig 4C and S3 Table). In contrast, northward movement was confirmed in 48 cases, with 46 individuals moving from Okinawa to Amami. Cases were most often seen in February and March in Okinawa, in the middle to the later stages of the breeding season (Tables 5 and 6, Fig 4C and S3 Table).

## Discussion

This is the first study to analyze the interchange and movement of humpback whales among four sighting regions in Japan. Although the matching results between the regions could have been underestimated owing to the accuracy of the automated matching system in this study (80.9%), the results of the photographic comparisons provide evidence that there are interchanges between the regions at some level, and these four areas are mainly utilized by the same population. Meanwhile, it should also be noted that the results from comparison between interchange indices and with-region return indices showed that there were fidelities to each region at some degree. Also, results showed that the frequency of interchanges varied among the three breeding regions in Japan. These findings suggest the possibility of existence of at least two groups in the population that utilized certain areas more often than the others. These results will contribute to the understanding of the detailed structure of the western North Pacific and will also provide important information for future conservation and management plans for this endangered population of humpback whales.

### Interchanges of humpback whales between the four regions

Among the four sighting areas in Japan, Hokkaido is the northernmost area located between Russia, the Eastern Bering Sea/Aleutians (feeding areas for humpback whales in the western North Pacific [39, 40]) and the breeding areas in Japan (Ogasawara, Amami, and Okinawa). These breeding areas are positioned 1,770, 2,100, and 2,340 km south of Hokkaido in a straight-line distance, respectively (Fig 1). Three out of the six whales found in Hokkaido were only observed in Okinawan waters in different years in this study. It has been suggested that humpback whales in Okinawa feed mainly off the Kamchatka Peninsula in Russia in summer [4], and their early arrival in Okinawa during the breeding season has been confirmed in December [17]. The discoveries of the same individuals in Hokkaido and Okinawa indicate that individuals which spend summer in the northern feeding areas migrate southward to Okinawa using the waters off the coast of Hokkaido as a corridor in autumn. Nevertheless, there is a possibility that the Hokkaido area is a feeding area for humpback whales, as its environmental conditions (such as latitude and water temperature) are similar to those of the known feeding areas, such as California [6]. However, the feeding behavior of humpback whales has never been reported in this area; thus, it is necessary to expand the survey area and the season in Hokkaido to obtain more data to verify this possibility. In contrast, no matches were found between Hokkaido and the other breeding areas of Ogasawara and Amami in this study. This is probably due to the very limited number of IDs obtained in Hokkaido in this study under the conditions of a relatively short survey period, and the lower sighting rate may have been caused by dense fog occurrence in this area during the survey period. Therefore, the interchange between Hokkaido and the two regions should be re-examined in future studies when the number of IDs in Hokkaido has increased.

Between the breeding areas of Amami and Okinawa, southward migration from Amami to Okinawa was more frequent in the first half of the breeding period, while northward migration from Okinawa to Amami was more frequent in the later half of the period within the same season (Fig 4C and S2 Table). For example, a male (‘A-98/R-411’) was first observed in Amami on February 17 and then found in Okinawa 7 d later on February 24 in a same year. The whale was later found in Okinawa on March 12 and 26, before moving north and observed again in Amami on April 3 (Table 7). A similar trend of southward movement in the earlier breeding season and northward movement in the later part of the season has also been observed between Okinawa and the Philippines [10]. Based on the reports of previous studies and the results of the current study, it can be concluded that humpback whales are likely to migrate from the feeding areas around the Kamchatka Peninsula in Russia and the Eastern Bering Sea/Aleutians, passing the coast of Hokkaido in autumn, and continue migrating to the south, stopping at the areas of Amami, Okinawa, and the Philippines along the islands. Conversely, at the end of the breeding season, they migrate northward stopping at the breeding areas on the way to the feeding ground. One male whale (‘A-15/R-198’), however, migrated south to Okinawa after being sighted in Amami, moved north toward Amami again, and then migrated south again toward Okinawa (Table 7). This suggests that whales do not necessarily pass through the areas, but also go back and forth between the areas during the breeding season. The utilization of multiple areas within a breeding season has also been observed in various regions of Hawaii [41] and Okinawa [42]. These results suggest the possibility that Amami and Okinawa are used as the same breeding area. On the other hand, it has been confirmed that the same individuals move between two other areas in Okinawa, Motobu and Kerama, much more frequently than between Amami and Okinawa in the same year [42]. Therefore, it is also necessary to re-examine whether Amami and Okinawa are used as the same breeding area after more data are obtained in the Amami region.

Between Amami and Ogasawara, one whale was confirmed to migrate from Ogasawara to Amami at the end of the same breeding season (Table 6, Fig 4A and S3 Table). Meanwhile, between Okinawa and Ogasawara, nine individuals were observed migrating from Okinawa to Ogasawara and two whales from Ogasawara to Okinawa, both in the early breeding season (Table 6, Fig 4B and S3 Table). These results indicate that humpback whales may not simply migrate in a north-south direction but also in an east-west direction to increase their chances of mating during the breeding season. Interestingly, the migration of whales between Okinawa and Ogasawara within the same season was confirmed to be five times more frequent from Okinawa to Ogasawara than in the opposite direction (Tables 5 and 6, Fig 4B and S3 Table). This suggests that many of the whales migrating from the feeding areas to Ogasawara are unlikely to move to the breeding areas in Amami and Okinawa, while a relatively larger number of whales that once migrated to those breeding areas also moved to Ogasawara within the same season. This could be related to the ocean currents that flow between the areas. The strong Kuroshio Current flows southwest to northeast along the coasts between Okinawa and Tokyo, and the Ogasawara Current flows from Tokyo to the south along the Izu-Ogasawara Arc. Therefore, it is conceivable that migration from Okinawa to Ogasawara is more likely to occur along these currents than migration in the opposite direction. However, this possibility needs to be further investigated by obtaining more data.

In this study, males were confirmed five times more frequently than females, in all the three breeding areas (Tables 3 and S1). At the same time, there were approximately twice as many males as females confirmed among the individuals that moved between multiple breeding areas in the same season (Table 5). These results further support the idea that males may move more actively between different ocean areas than females, to obtain more mating opportunities [41, 42]. It should be noted, however, that males are more behaviorally identifiable than females. The probability of fluke-up dives [29, 35], and male-biased sex segregation of humpback whales in breeding grounds have been noted in previous studies [29, 43]. Therefore, it is possible that the migration of males was confirmed more often than that of females, in this study. Meanwhile, females were also observed migrating from Ogasawara to Amami, and between Okinawa and Ogasawara within the same season (Tables 5 and 6). Although the number of cases confirmed for females was much smaller than that for males, it suggests that both males and females may actually utilize multiple areas during the breeding season to increase their mating opportunities. Since approximately half of the individuals confirmed in multiple areas were of unknown sex, the results of sex determination by genetic analysis should also be conducted to analyze the actual trend of migration in both sexes between areas.

The days between sightings in different Japanese breeding areas within same years was documented for the first time in the present study. The duration of the migration between Ogasawara and Amami within the same season was 16 d. Between Ogasawara and Okinawa, the duration between the observations among the areas ranged from 21–100 d, with an average of 42.3 d. In the case between Amami and Okinawa, the duration was in between 4–69 d, with an average of 16.9 d (Table 5). However, since the present migration period is based on the first and last observation dates in the two areas, the days for the migration of the whales between areas may have overestimated. Therefore, a detailed investigation of the migration route and the actual days of migration between the breeding areas will require verification using continuous spatiotemporal data and satellite-tagging surveys. Furthermore, future studies on the relationship between the newly confirmed potential migratory corridors in Japanese waters [23–25] and the known feeding and breeding areas will be useful in clarifying the actual migratory routes of the populations.

Although the analysis of the interchange indices and movement between the areas revealed the frequency of interchanges among the sighting areas in Japanese waters, it should be noted that the interchange between the regions were varied, and the within-region return indices for each breeding area were higher than the interchange indices between other regions. In a previous study, the interchange and within-region return indices between the three islands in Hawaii were compared, and both the interchange and within-region return indices for all areas ranged between 0.2 and 0.3 [6]. Therefore, the authors suggested that the same population utilized the three different areas equally [6]. In contrast, the interchange indices among the main land of Mexico, Revillagigedos Archipelago, and Baja were approximately 0.2, while the respective within-region return indices to each area were more than three times higher, ranging from 0.9–1.3. Therefore, it was suggested that separate subpopulations with high site fidelity to each region utilized the breeding areas in the eastern North Pacific populations [6].

In the results of our study, the within-region return indices of the waters other than Hokkaido, where there was no resight of the same individual, were all 1.5 to 2 times higher than the interchange indices between other regions (Tables 2 and 4). The results suggest that whales in Japanese waters also exhibit some degree of fidelity to their respective breeding areas. This further supports a similar statement in Acebes et al. [10] that the breeding areas in the western North Pacific show site fidelity to each area.

### Different migration patterns along with geographical features

**Fig 5:**
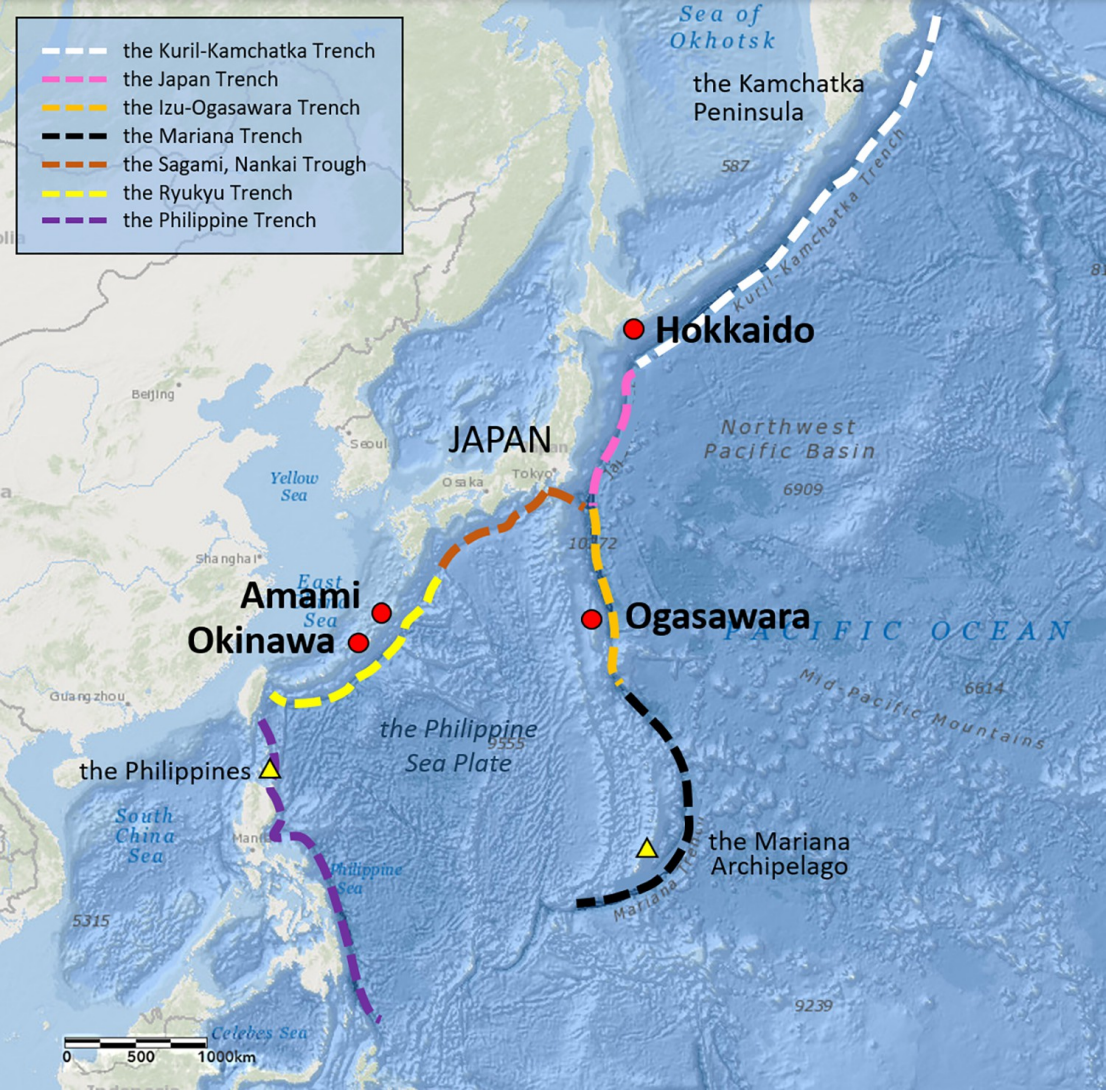
Sighting areas of humpback whales (*Megaptera novaeangliae*) among the western North Pacific DPS along the trenches between the feeding area and breeding areas. Red circles represent the sighting areas in this study. The map image is published from PLOS ONE under a CC BY license, with permission from Environmental Systems Research Institute, Inc. (Esri), original copyright 2022, also the content is the intellectual property of Esri and is used herein with permission. Copyright © 2022 Esri and its licensors. All rights reserved.

The three breeding areas; Ogasawara, Amami, and Okinawa are located on two different island arcs: the Izu-Ogasawara arc and the Ryukyu arc, which are positioned along the two different trenches; both lie from north to south at the east and west ends of the Philippine Sea Plate (Fig 5).

Amami and Okinawa are both located on the Ryukyu Arc along the Ryukyu Trench, which runs from the Pacific coast of Kyushu to the Okinawa area and are connected to the Philippine Trench (Fig 5). Ogasawara, on the other hand, is located on the Izu-Bonin Arc along the Izu-Ogasawara Trench, which leads from Tokyo to the Ogasawara Islands and is connected to the Mariana arc, the Mariana Trench in the south.

In this study, the interchange between Amami and Okinawa was much more active compared to the interchanges between Ogasawara and Amami, or between Ogasawara and Okinawa (Table 2). In addition, the number of within-season movements of whales between Ogasawara and both Amami and Okinawa were much smaller compared to the numbers between Amami and Okinawa (Table 5). These results suggest that the interchanges between the three breeding areas vary. Previous studies have shown that humpback whales tend to use and follow geographical features, such as islands and seamounts, during migration [44, 45]. Therefore, based on the results of the interchange index, within seasonal movements, and site fidelity to each regions as well as the geographical features between the three regions, it can be inferred that the humpback whales utilizing the breeding areas around Japan may be divided into two smaller groups: one tend to migrate mainly to Amami and Okinawa along the Ryukyu island beside the Ryukyu Trench, and the other to Ogasawara, which tend to migrates south along the Izu-Ogasawara island beside the Izu-Ogasawara Trench.

The existence of different groups along the different trenches confirmed in this study may be common not only to Japanese waters but also to the entire western North Pacific stock. The number of individuals observed between Okinawa and the Philippines, located on the Philippine Trench leading south of the Ryukyu Trench, is higher than that between the Philippines and the Ogasawara Islands [10] (Table 2 and Fig 5). The number of the same individuals confirmed between Ogasawara and the Mariana Islands, which is located on the Mariana Trench extending south from the Ogasawara Trench, was higher than that between Mariana and Okinawa or the Philippines [11] (Table 2 and Fig 5). Moreover, there was no correlation between the distance between breeding areas and the value of the interchange index in this study. This result also suggests that perhaps the degree of interchange between regions is not based on simple linear distances but may be related to geographical factors between the areas. In addition, there were significant differences in mtDNA haplotype frequencies reported between Mariana and both the Philippines and Okinawa, while no significant differences were confirmed between Mariana and Ogasawara [11, 16]. The two potentially different groups with distinct migration patterns, confirmed in this study—one which primarily utilizes the breeding areas of Amami, Okinawa, and the Philippines, and migrates southward along the Ryukyu and Philippines Trenches, and the other which primarily utilizes the Ogasawara and Mariana Archipelago breeding areas and migrates southward along the Izu-Ogasawara and Mariana Trenches—further support the thesis in previous studies that there might be several different groups in the western North Pacific populations [11]. However, the DNA analysis comparison between the regions should be conducted with increased sample size in the future to determine the existence of several sub groups in the western North Pacific region.

Also, some of the interchange indices in this study were calculated with relatively small number of IDs, such as in Hokkaido and Mariana. Therefore, more detailed analysis on the relationship between interchange frequency and distances between the regions need to be conduct when more data is available for these areas. Moreover, it is also important to conduct surveys using satellite tags in order to understand the actual migration route of the whales.

## Conclusion

In this study, the results from the photo matching comparison among several sighting areas in Japan indicate that Japanese waters are used by a common population of humpback whales. Furthermore, the results also suggest that there may be at least two smaller groups within the population that have different migration patterns, possibly based on geographic characteristics, and they may be intricately interrelated as they move between regions. The genetic comparisons among the regions with the latest samples are needed to evaluate long-term interchanges among the areas to examine the degree of segregation in each sighting region in this population. In addition, it is also useful to conduct song comparisons between waters as a validation of short-term interchanges between breeding areas, as suggested by Darling et al. [46, 47]. Satellite-tagging surveys on humpback whales to elucidate the actual migration route between the regions and to understand the exact migration timing within the same season are needed. Finally, international joint research and collaboration involving all organizations conducting research in the western North Pacific is crucial to conduct appropriate conservation management of this endangered humpback whale population.

## Supporting information

S1 Table Years of observation of humpback whales which observed in three different breeding areas (Ogasawara, Amami, and Okinawa) in the different years. (XLSX)

S2 Table Distance (km), interchange, and standardized difference in interchange indices between sighting areas of humpback whales in the western North Pacific. (XLSX)

S3 Table Directions and months of within-season movements between Ogasawara, Amami, and Okinawa. (XLSX)

## References

[1] Dawbin WH. The seasonal migratory cycle of humpback whales. In: Norris KS, Editor, Whales, Dolphins, and Porpoises. Berkeley, CA, University of California Press; 1966. pp. 145–170. doi: 10.1525/9780520321373-011

[2] Clapham PJ, Mead JG. *Megaptera novaeangliae*. Mammalian Species. 1999; 604: 1–9. doi: 10.2307/3504352

[3] Calambokidis J, Falcone EA, Quinn TJ, Burdin AM, Clapham, PJ, Ford JKB, et al. SPLASH: Structure of Populations, Levels of Abundance and Status of Humpback Whales in the North Pacific. Final report for Contract AB133F-03-RP-00078. For U.S. Dept of Commerce Western Administrative Center Seattle, Washington. 2008.

[4] Titova OV, Filatova OA, Fedutin ID, Ovsyanikova EN, Okabe H, Kobayashi N, et al. Photo-identification matches of humpback whales (*Megaptera novaeangliae*) from feeding areas in Russian Far East seas and breeding grounds in the North Pacific. Mar Mamm Sci. 2018; 34: 100–112. doi: 10.1111/mms.12444

[5] Urban RJ, Aguayo LA. Spatial and seasonal distribution of the humpback whale, *Megaptera novaeangliae*, in the Mexican Pacific. Mar Mamm Sci. 1987; 3: 333–344. doi: 10.1111/j.1748-7692.1987.tb00320.x

[6] Calambokidis J, Steiger GH, Straley JM, Herman LM, Cerchio S, Salden DR, et al. Movements and population structure of humpback whales in the North Pacific. Mar Mamm Sci. 2001; 17: 769–794. doi: 10.1111/j.1748-7692.2001.tb01298.x

[7] Mori K, Sato F, Yamaguchi M, Suganuma H, Ueyanagi S. Distribution, migration, and local movements of humpback whale (*Megaptera novaeangliae*) in the adjacent waters of the Ogasawara (Bonin) Islands, Japan. PhD thesis, Tokai University. 1998; 45: 197–213.

[8] Tsujii K, Akamatsu T, Okamoto R, Mori K, Mitani Y, Umeda N. Change in singing behavior of humpback whales caused by shipping noise. PLoS ONE. 2018; 13:e0204112. doi: 10.1371/journal.pone.0204112 30356328 PMC6200181

[9] Kobayashi N, Okabe H, Kawazu I. Higashi N, Miyahara H, Kato H, et al. Spatial distribution and habitat use patterns of humpback whales in Okinawa, Japan. Mamm Stud. 2016a; 41: 207–214. doi: 10.3106/041.041.0405

[10] Acebes JMV, Okabe H, Kobayashi N, Nakagun S, Sakamoto T, Hirney B, et al. Interchange and movements of humpback whales (*Megaptera novaeangliae)* between western North Pacific winter breeding grounds in northern Luzon, Philippines and Okinawa, Japan. J Cetacean Res Manage. 2021; 22 (1): 39–53. doi: 10.47536/jcrm.v22i1.201

[11] Hill MC, Bradford AL, Steele D, Baker CS, Ligon AD, Ü AC, et al. 2020. Found: a missing breeding ground for endangered western North Pacific humpback whales in the Mariana Archipelago. Endang Species Res. 2020; 41: 91–103. doi: 10.3354/esr01010

[12] Nishiwaki M. Ryukyuan whaling in 1961. Scientific Reports. Whale Research Institute. 1961; 16: 19–28.

[13] Barlow J, Calambokidis J, Falcone EA, Baker CS, Burdin AM, Clapham PJ, et al. Humpback whale abundance in the North Pacific estimated by photographic capture-recapture with bias correction from simulation studies. Mar Mamm Sci. 2011; 27: 793−818. doi: 10.1111/j.1748-7692.2010.00444.x

[14] NOAA. Endangered and threatened species: Identification of 14 distinct population segments of the humpback whale (*Megaptera novaeangliae*) and revision of species-wide listing. Fed Regist. 2016; 81:62260−62320.

[15] Cooke JG. *Megaptera novaeangliae*. The IUCN Red List of Threatened Species. 2018; e.T13006A50362794. doi: 10.2305/IUCN.UK.2018-2.RLTS.T13006A50362794.en

[16] Baker CS, Steel D, Calambokidis J, Falcone EA, Gozález-Peral U, Barlow J, et al. Strong maternal fidelity and natal philopatry shape genetic structure in North Pacific humpback whales. Mar Ecol Prog Ser. 2013; 494: 291−306. doi: 10.3354/meps10508

[17] Uchida S, Higashi N, Maeda H, Koido T, Takemura A. What is a Humpback Whale? Kings of the Sea: Humpback Whales II: A 1999–2005 Survey of Cetaceans in the Japanese Waters. The UFJ Environment Foundation. 2005. pp. 22.

[18] Acebes JV, Darling JD, Yamaguchi M. Status and distribution of humpback whales (*Megaptera novaeangliae*) in northern Luzon, Philippines. J Cetacean Res Manage. 2007; 9: 37–43.

[19] Kobayashi N, Okabe H, Kawazu I, Higashi N, Miyahara H, Kato H, et al. Peak mating and breeding period of the humpback whale (*Megaptera novaeangliae*) in Okinawa Island, Japan. Open J Animal Sci. 2016b; 6: 169–179. doi: 10.4236/ojas.2016.63022

[20] Darling JD, Mori K. Recent observations of humpback whales (*Megaptera novaeangliae*) in Japanese waters off Ogasawara and Okinawa. Can J Zoo–Revue Canadienne de Zoologie. 1993; 71(2): 325–33. doi: 10.1139/Z93-045

[21] Nakagun S, Smoll LI, Sato T, Layusa CAA, Acebes JMV. Interchange of humpback whales (*Megaptera novaeangliae*) between northern Philippines and Ogasawara, Japan, has implications for conservation. Pac. Conserv. Biol. 2020; 26: 378–83. doi: 10.1071/PC19003

[22] Okabe H, Acebes JMV, Kobayashi N, Nakagun S, Higashi N, Uchida S. To go or not to go: Movements of humpback whales between breeding grounds in Okinawa, Japan, and the Philippines. In: Proceedings of the 22nd Biennial Conference on the Biology of Marine Mammals. Halifax, Canada. 2017.

[23] Kogi K. Can you see them in Mikura Island waters? Cetaceans other than Indo-Pacific bottlenose dolphin. 2015 May 23 [cited Sep 13, 2021]. In: Ocean α [Internet]. Tokyo, Japan: © 2020 oceana All rights reserved. [about 2 screens]. [In Japanese].https://oceana.ne.jp/column/56455

[24] Katsumata T, Hirose A, Nakajo K, Shibata C, Murata H, Yamakoshi T et al. Evidence of winter migration of humpback whales to the Hachijo island, Izu archipelago off the southern coast of Tokyo, Japan. Cetacean Popul. Stud. 2021; 3:164−174.

[25] Takada N. Result of the surveys on whales in the 2021 season. 2021 May [cited Sep 13, 2021]. In: Yakushima Whale & Dolphin Research Center [Internet]. Yakushima, Japan: Yakushima Whale & Dolphin Research Center. [about 2 screens]. [In Japanese]. https://www.yakushima-whale.com/

[26] Rasmussen K, Palacios DM, Calambokidis J, Saborío MT, Dalla Rosa L, Secchi ER et al. Southern Hemisphere humpback whales wintering off Central America: Insights from water temperature into the longest mammalian migration. Biol Lett. 2007; 3(3):302−5. doi: 10.1098/rsbl.2007.0067 17412669 PMC2390682

[27] Tyack P, Whitehead H. Male competition in large groups of wintering humpback whales. Behaviour. 1983; 83: 132−154. doi: 10.1163/156853982X00067

[28] Clapham PJ, Palsbøll PJ, Matilla DK, Vasquez O. Composition and dynamics of humpback whale competitive groups in the West Indies. Behaviour. 1992; 122: 182−194. doi: 10.1163/156853992X00507

[29] Craig AS, Herman LM. Habitat preferences of female humpback whales *Megaptera novaeangliae* in Hawaiian waters are associated with reproductive status. Mar Ecol Prog Ser. 2000; 193: 209−216. doi: 10.3354/meps193209

[30] Mitani Y, Kobayashi N, Okabe H. The North-South migration of humpback whales: Photo-identification match of an individual from the Pacific coast of eastern Hokkaido and breeding areas in Okinawa. Mammalian Sci. 2020; 60 (2): 170. doi: 10.11238/mammalianscience.60.170

[31] Darling JD, Gibson KM, Silber GK. Observations on the abundance and behavior of humpback whales (*Megaptera novaeangliae*) off West Maui, Hawaii, (1977–1979). In: Payne R, Editor. Communication and Behavior of Whales. AAAS Selected Symposia Series. Westview Press, Boulder; 1983. pp. 201−222.

[32] Glockner DA. Determining the sex of humpback whales (*Megaptera novaeangliae*) in their natural environment. In: Payne RS, Editor. Behavior and Communication of Whales. Westview Press, Boulder, CO; 1983. pp. 447–464.

[33] Katona SK, Whitehead HP. Identifying humpback whales using their natural markings. Polar Record. 1981; 20: 439–444. doi: 10.1017/S003224740000365X

[34] Yoshikawa T, Hida M, Lee C, Okabe H, Kobayashi N, Ozawa S et al. Identification of over one thousand individual wild humpback whales using fluke photos. In Proceedings of the 17th International Joint conference on Computer Vision, Imaging and Computer Graphics Theory and Applications. 2022. 4: ISSN2184-4321, 957–967.

[35] Garrigue C, Aguayo A, Amante-Helwig VLU, Baker CS, Caballero P, Clapham P, et al. Movements of humpback whales in Oceania, South Pacific. J Cetacean Res. Manage. 2002; 4(3): 255–60.

[36] Urban RJ, Jaramillo LA, Auayo LA, Ladron de Guevara PP, Salinas ZM, Alvares FC et al. Migratory destinations of humpback whales wintering in the Mexican Pacific. J Cetacean Res Manage. 2016; 2 (2): 13–22.

[37] Darling JD, Cerchio S. Movement of a humpback whale (*Megaptera novaeangliae*) between Japan and Hawaii. Mar Mamm Sci. 1993: 9384–89.

[38] Salden DR, Herman LM, Yamaguchi M, Sato F. Multiple visits of individual humpback whales (*Megaptera novaeangliae*) between the Hawaiian and Japanese winter grounds. Canadian Journal of Zoology. 1999; 77: 504–508.

[39] Nishiwaki M. Distribution and migration of the larger Cetaceans in the North Pacific as shown by Japanese whaling results. In: Norris K, editor. Whales, Dolphins and Porpoises. Univ. Calif. Press. 1966. pp. 171–191.

[40] Ohsumi S, Masaki Y. Japanese whale marking in the North Pacific, 1963–1972. J. Fish. Res. Board Can. 1975: 12, 171–219.

[41] Cercio S, Gabriele CM, Norris TF, Herman LM. Movement of humpback whales between Kauai and Hawaii: Implications for population structure and abundance estimation in the Hawaiian Islands. Mar. Ecol. Prog. Ser. 1998; 175: 13–22. doi: 10.3354/meps175013

[42] Kobayashi N, Okabe H, Kawazu I, Higashi N, Kato K, Miyahara H, et al. Distribution and local movement of humpback whales in Okinawan waters depend on sex and reproductive status. Zool. Sci. 2017; 34: 58–63. doi: 10.2108/zs160012 28148212

[43] Brown MR, Corkeron PJ, Hale PT, Shultz KW, Bryden MM. Evidence for a sex-segregated migration in the humpback whale (*Megaptera novaeangliae*). Proc R Soc Lond B Biol Sci. 1995. 259:229–234. 10.1098/rspb.1995.0034 7732039

[44] Garrigue C, Clapham PJ, Geyer Y, Kennedy AS, Zerbini AN. Satellite tracking reveals novel migratory patterns and the importance of seamounts for endangered South Pacific humpback whales. Royal Society Open Sci. 2015; 2:150489. doi: 10.1098/rsos.150489 26716006 PMC4680621

[45] Thouless C. Where the Whales Go: The Migration Routes of Humpbacks in the South West Atlantic. M. Sc. Thesis, The University of California, San Diego. 2021. Available from: https://escholarship.org/uc/item/1693m1b5

[46] Darling JD, Acebes JMV, Yamaguchi M. Similarity yet a range of differences between humpback whale songs recorded in the Philippines, Japan, and Hawaii in 2006. Aquat. Biol. 2014; 21: 93–107. doi: 10.3354/ab00570

[47] Darling JD, Acebes JMV, Frey O. Urbán RJ, Yamaguchi M. Convergence and divergence of songs suggests ongoing, but annually variable, mixing of humpback whale populations throughout the North Pacific. Sci Rep. 2019; 9: 7002. doi: 10.1038/s41598-019-42233-7 31065017 PMC6505537

